# Structure-guided modification and optimization of antibody VRC07

**DOI:** 10.1186/1742-4690-9-S2-O34

**Published:** 2012-09-13

**Authors:** Y Kwon, I Georgiev, S O'Dell, W Shi, G Chuang, Y Yang, B Zhang, J Zhu, GJ Nabel, JR Mascola, PD Kwong

**Affiliations:** 1 National Institutes of Health, Kensington, MD, USA

## Background

Eliciting a neutralizing human antibody against HIV-1 still remains to be elusive. Nevertheless, a number of studies have reported isolation of potent and broadly reactive antibodies against HIV-1 from HIV-1 infected patient serum. Antibody VRC01 is one of these kinds that binds to the CD4 binding site of gp120 and neutralizes the viruses. Recently, we identified antibody VRC07, which is more potent and broadly reactive anti-HIV-1 antibody than its derivative, VRC01.

## Methods

In this study, we determined the crystal structure of gp120 in complex with VRC07 and utilized mechanistic insights and structure-guided modification to increase potency and breadth.

## Results

A four amino acid insertion in the CDR H3 loop of VRC07 provided for more extensive contacts with gp120 than with VRC01. The structure also revealed that residue Gly54 of VRC07 could be replaced with amino acids with bulky side chains to mimic residue Phe43 of CD4. Indeed, all VRC07 variants, in which Gly54 was replaced with Arg, Leu, Phe, Trp, or Tyr, showed enhanced affinity to a panel of different HIV-1 gp120s. Furthermore, most of these Gly54 alterations showed enhanced potency and breadth against a panel of clade B and C viruses in TZM-bl cell-based neutralization assay. Crystal structures of gp120 in complexes with these VRC07 Gly54 variants confirmed that their side chains mimicked Phe43 of CD4. Computational analysis of the VRC07-gp120 interface in the crystal structure identified residues Ile30 and Ser58 as likely targets for improvement (with Gln and Asn, respectively). These changes introduced additional hydrogen bonds to the VRC07-gp120 interfaces and further enhanced VRC07 potency.

## Conclusion

Thus, our optimization of antibody VRC07 demonstrated that a structure-guided approach can be used to increase both antibody potency and breath. The optimized VRC07 developed here can be used as the basis for further structure-guided improvement or for optimization via in vitro selection.

